# Multiple-kernel learning for genomic data mining and prediction

**DOI:** 10.1186/s12859-019-2992-1

**Published:** 2019-08-15

**Authors:** Christopher M. Wilson, Kaiqiao Li, Xiaoqing Yu, Pei-Fen Kuan, Xuefeng Wang

**Affiliations:** 1 Department of Biostatistics and Bioinformatics at Moffitt Cancer Center, Tampa, FL USA; 2 Department of Applied Mathematics and Statistics at Stony Brook University, Stony Brook, NY USA

**Keywords:** Classification, Multiple kernel learning, Genomics, Data integration, Machine learning, Kernel methods

## Abstract

**Background:**

Advances in medical technology have allowed for customized prognosis, diagnosis, and treatment regimens that utilize multiple heterogeneous data sources. Multiple kernel learning (MKL) is well suited for the integration of multiple high throughput data sources. MKL remains to be under-utilized by genomic researchers partly due to the lack of unified guidelines for its use, and benchmark genomic datasets.

**Results:**

We provide three implementations of MKL in R. These methods are applied to simulated data to illustrate that MKL can select appropriate models. We also apply MKL to combine clinical information with miRNA gene expression data of ovarian cancer study into a single analysis. Lastly, we show that MKL can identify gene sets that are known to play a role in the prognostic prediction of 15 cancer types using gene expression data from The Cancer Genome Atlas, as well as, identify new gene sets for the future research.

**Conclusion:**

Multiple kernel learning coupled with modern optimization techniques provides a promising learning tool for building predictive models based on multi-source genomic data. MKL also provides an automated scheme for kernel prioritization and parameter tuning. The methods used in the paper are implemented as an R package called RMKL package, which is freely available for download through CRAN at https://CRAN.R-project.org/package=RMKL.

**Electronic supplementary material:**

The online version of this article (10.1186/s12859-019-2992-1) contains supplementary material, which is available to authorized users.

## Background

### Motivation

Data integration is an emerging topic of interest in cancer research. Making decisions based upon metabolomic, genomic, etc. data sources can lead to better prognosis or diagnosis than using clinical data alone. Though data sources may have different background noise levels, formats, and biological interpretations, a framework for integrating data of similar and heterogeneous types has been proposed [1]. Classification or prediction based on data from a single high throughput source may require machine learning techniques since the number of genes or metabolites will inevitably be larger than the number of samples. Both supervised and unsupervised machine learning methods have been successfully utilized for classification [2–4], regression [5, 6], and identification of latent batch effects [7, 8]. In this paper, we focus on supervised classification of dichotomized survival outcome for various cancer types, specifically discussing support vector machines and multiple kernel learning.

### Support vector machines

Support vector machines (SVMs) were originally proposed to find a hyperplane such that two classes of data are on different sides of the hyperplane and have the maximal distance between the two classes. There have been several improvements presented for SVM such as adding additional constraints to the optimization problem that allow the problem to be feasible when the two classes are not perfectly separable. An additional term is added to the objective function that penalizes misclassified samples, this resulting formulation is known as soft-margin [9].

A second major improvement to SVM is applying the kernel trick to allow for a non-linear classification rule. Kernel functions are used to provide different similarity measures between samples. The correlation (dot product) matrix is used to find a linear classifier. Other common kernels are polynomial kernels and radial kernels for continuous features [10]. Moreover, kernels have been proposed for nominal and ordinal data, hence we can construct kernels based on demographic characteristics (race, gender, height, age, etc.) [11]. Below are formulas for different similarities between two samples *x* and *y*:

1a $$\begin{array}{*{20}l} &\bullet\text{ Linear:} K(x,y)=< x,y>=x^{T}y, \end{array} $$

1b $$\begin{array}{*{20}l} &\bullet\text{ Polynomial:} K(x,y)=(\nu< x,y> + offset)^{a}, \end{array} $$

1c $$\begin{array}{*{20}l} &\bullet\text{ Radial:} K(x,y)=\exp(-\sigma||x-y||^{2}/2), \end{array} $$

1d $$\begin{array}{*{20}l} &\bullet\text{ Clinical nominal:} K(x, y) = \left\{\begin{array}{ll} 1, & \text{if} x=y, \\ 0, & \text{if} x\neq y, \end{array}\right. \end{array} $$

1e $$\begin{array}{*{20}l} &\bullet\text{ Clinical ordinal:} K(x,y)=\frac{r-|x-y|}{r}, \end{array} $$

where *a* is the degree and *ν* is the coefficient of the highest order term of an *a* degree polynomial, *σ* controls smoothness of the decision boundary for a radial kernel, and *r* is the range of the ordinal levels. We use the parameterization found in the kernlab R package for the linear, polynomial and radial kernels [12].

Kernel methods are attractive because they do not make parametric assumptions to construct a model, for instance, these methods are not sensitive to outliers and are distribution-free [13]. Unfortunately, the solutions can be sensitive to the choice of parameter and there is no universal best set of parameters for a given data type. Typically, cross-validation is used to identify the parameter that provides the highest prediction accuracy. Ultimately, there may not be one single optimal kernel, but a combination of kernels may provide a better classifier than a single kernel. It can be shown that the sum, product, and convex combination of kernels yields another kernel [14]. This leads to an opportunity to construct a classifier using a convex combination of candidate kernels.

### Multiple kernel learning

Multiple kernel learning (MKL) algorithms aim to find the best convex combination of a set of kernels to form the best classifier. Many algorithms have been presented in recent years and they form two classes. First, wrapper methods solve MKL by first solving a single SVM problem for a given set of kernel weights, and then they update the kernel weights. Since wrapper functions rely on solving SVM, they appeal to existing well-developed solvers, thus they can be relatively easy to implement. The second class of MKL algorithms utilize more sophisticated optimization methods to greatly reduce the number of SVM computations, allowing for them to solve the problem with a much larger number of kernels than wrapper methods. We will focus on two wrapper methods (SimpleMKL [15], Simple and Efficient MKL (SEMKL) [16]), as well as, and an example of a second class of MKL algorithms DALMKL [17].

Sparse MKL solutions do not typically outperform uniformly weighted kernels [18]. There is still great value in sparse kernel weights, specifically, the model can be easier to interpret with fewer non-zero kernel weights. Each MKL method can provide an ordering for the importance of a data type or features that may prompt investigators towards data sources that contain the most relevant information for classification. Ranking data sources can help researchers focus their studies on gene/metabolite sets or the data types that are most likely to lead to meaningful results.

Several studies have applied MKL to genomic data. An extensive comparison of regression techniques, including support vector regression (SVR) and Bayesian multitask MKL, has been conducted to predict drug sensitivity using six genomic, epigenomic, and proteomic profiling datasets for human breast cancer cell lines [19]. Bayesian multitask MKL involves the selection of priors and different selection of priors can lead to a dramatically different result. MKL was also implemented to predict survival at 2000 days from diagnosis, using the METABRIC dataset for breast cancer, and observed that predictive accuracy can be increased by grouping genes within a pathway into a single kernel [20]. These papers illustrate that MKL can be effectively applied to data that is from multiple sources and how it can be used to analysis high dimensional data, however, MKL remains an under-utilized tool for genomic data mining. This article aims to bridge these gaps by providing a unified survey of MKL methodology, highlighting its unique benefits in tackling challenges in large-scale omics data analysis, and establishing benchmarked models for further algorithm development.

This paper is organized as follows. “Implementation” section discusses practical issues when conducting MKL, and describes the features offered our package RMKL. “Results” section describes the results from one experiment which uses simulated data, and two experiments that use real data from The Cancer Genome Atlas (TCGA). Lastly, in “Conclusion” section, we make observations regarding our results and mention several areas for future work.

## Implementation

SimpleMKL uses subgradient descent to find the direction has the most improvement. Then it uses lines search to find the optimal set of kernel weights. For each candidate set of kernel weights, SimpleMKL must solve an SVM problem iteratively along the vector of maximum improvement. SEMKL can decrease the computational burden dramatically by updating the set of kernel weights with an explicit formula derived using the Cauchy-Schwarz inequality as opposed to using line search.

DALMKL optimizes the dual augmented Lagrangian of a proximal formulation of the MKL problem. This formulation presents a unique set of problems such as the conjugate of a loss function must have no non-differentiable points in the interior of its domain and cannot have a finite gradient at the boundary of its domain. The inner function is differentiable and the gradient and Hessian only depend on the active kernels making gradient descent efficient. DALMKL is written in C++, and uses Newton descent to update the kernel weights. A flowchart describing how general wrapper methods and DALMKL are implemented can be found in Additional file 1: Figure S2.

There are many considerations that must be made before conducting SVM or any MKL algorithm. One of the most important is the prioritization of features and kernels. Even though SVM does not deal with each feature directly it can suffer from the curse of dimensionality. If there are a large number of features and a very small number of features can separate the data, then SVM will not necessarily find the best subspace that separates the data. Feature prioritization can improve the accuracy of SVM [10, 20]. Features can be prioritized by determining which features have the biggest effect size or smallest p-value from a *t*-test or more robust two group comparisons such as the Wilcoxon rank-sum test.

**Fig. 1:**
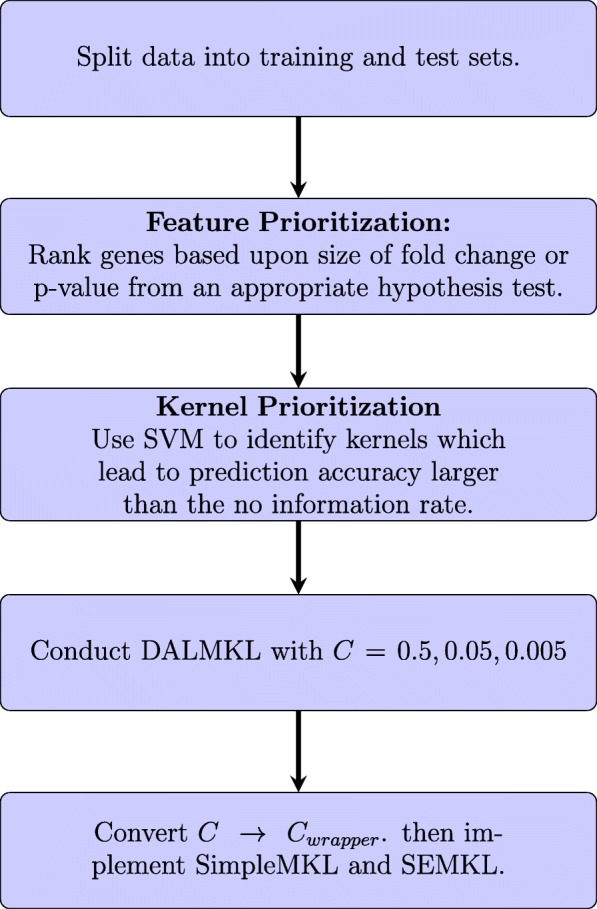
Recommended workflow for an MKL experiment

Kernel prioritization is important to alleviate many potential problems for MKL. For instance, if many kernels share a lot of redundant information then the efficiency of MKL can greatly diminish since many wrapper methods seek a sparse combination of kernels. Kernels that can both classify the data and yet provide different boundaries, similar to ensemble learning. A potential method for prioritizing kernels is to conduct SVM with each candidate kernel, then determine the kernels with the largest accuracy or eliminate the kernels with accuracy lower than the no information rate. Figure 1 summarizes the workflow we use and recommend for the implementation of MKL. There has been work using minimal redundancy maximal relevance criteria, and kernel alignment to remove kernels that share too similar [21].

We present an R package, RMKL, which can implement cross-validation for training SVM and support vector regression models, as well as MKL for both classification and regression problems. Our package is equipped with implementations of SimpleMKL, SEMKL, and DALMKL under two loss functions. We demonstrate each of these three implementations in simulated and real data to compare their performance. RMKL is freely available for download through CRAN at https://CRAN.R-project.org/package=RMKL. Next, we further discuss the features of RMKL.

There are several features in RMKL that aim to make the implementation of MKL easier. For instance, we provide a wrapper function to compute kernel matrices which can provide kernels for training and test set. Another convenient function in RMKL is a wrapper function for conducting cross-validation for SVM. A challenge of MKL wrapper methods is that there are no guidelines for selecting the penalty parameter. Fortunately, there are recommended values for the penalty parameter (0.5, 0.05, and 0.005) for DALMKL. Unfortunately, direct comparisons between SimpleMKL, SEMKL, and DALMKL are not possible using the same cost parameter in all three implementations. We provide a function that uses the solution of DALMKL to estimate for a comparable cost parameter for SimpleMKL and SEMKL.

## Results

### Benchmark example

In addition to accuracy, an important characteristic of MKL is the learning of kernel weights. In this example, 9 datasets are generated with two groups and the amount overlap between the two groups varies. The two groups have 50 observations from a bivariate normal distribution where the mean of group 1 was fixed at (5,5), and the means of group 2 were {(-4,-4),(-3,-3), …, (4,4)}. The covariance structure of the two groups were

$$\begin{array}{*{20}l} \Sigma_{1}= \ \left[ \begin{array}{cc}1 & 0\\ 0 & 1\end{array}\right], \text{ and }\Sigma_{2}= \ \left[ \begin{array}{cc}1 & -0.5\\ -0.5 & 1\end{array}\right]. \end{array} $$

If the two groups do not overlap, then we expect a radial kernel with a small scale parameter to have a larger weight than a radial kernel with a larger scale parameter (see kernel parameterization in the kernlab R package), leading to a smooth boundary. On the other hand, if there is a large amount of overlap between the groups, we expect lower accuracy and a less smooth classification rule. Thus a larger scale hyperparameter should be preferred.

**Fig. 2:**
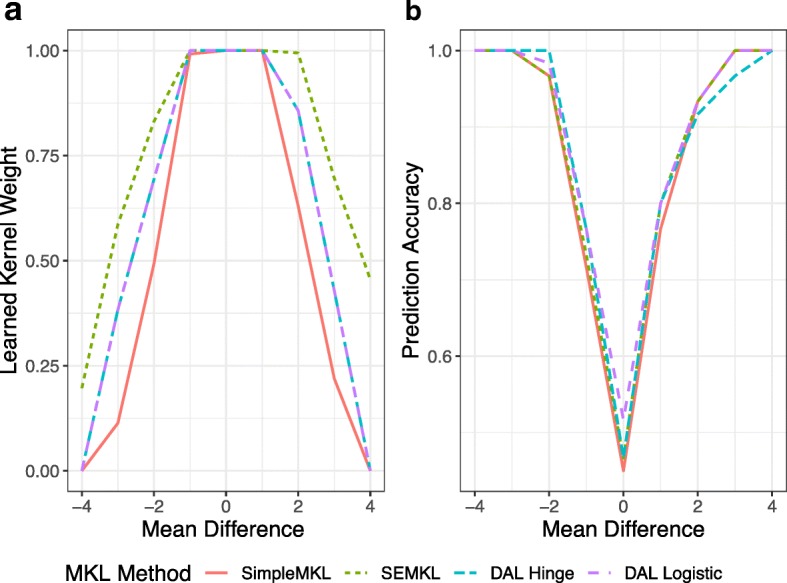
Results from SEMKL, SimpleMKL, and DAMKL on 9 benchmark datasets, where two radial kernels *K*_1_ and *K*_2_ with *σ*_1_=2 and *σ*_2_=0.05 were used. **a** Displays the learned kernel weight of *K*_1_ as the mean of each group changes. **b** Displays the predictive accuracy of each algorithm as the distance between each group changes. DAL Hinge and DAL Logistic refer to conducting DALMKL under different loss functions

We consider two radial kernels, denoted *K*_1_ and *K*_2_, with hyperparameters *σ*_1_=2 and *σ*_2_=0.04. In Fig. 2a, notice that as the amount of overlap between the two groups increases the weight for *K*_1_ increases. This yields a classification rule that is less smooth and can accommodate for the overlapping groups. When there is little overlap between the groups, we see that *K*_2_ is given much more weight than *K*_1_, leading to a smooth classification rule for perfectly separable data All algorithms can classify perfectly when there is no overlap, but when the groups are completely overlapping, the prediction accuracy of each algorithm is approximately 0.5 (Fig. 2b).

### TCGA ovarian

Bell et al. (2011) provide integrative analyses of The Cancer Genome Atlas (TCGA) ovarian cancer dataset [22]. Survivorship for ovarian cancer is difficult to predict from clinical information only, which is limited since most cancers are late stage. Information from high throughput data sources can be utilized to increase prediction accuracy. To illustrate MKL as a data integration tool, we perform MKL to find the best kernel for clinical and miRNA gene expression data separately, and then combine them into a single analysis. Our goal is to predict if a patient will live longer than three years after diagnosis and patients who were right-censored were not considered.

**Fig. 3:**
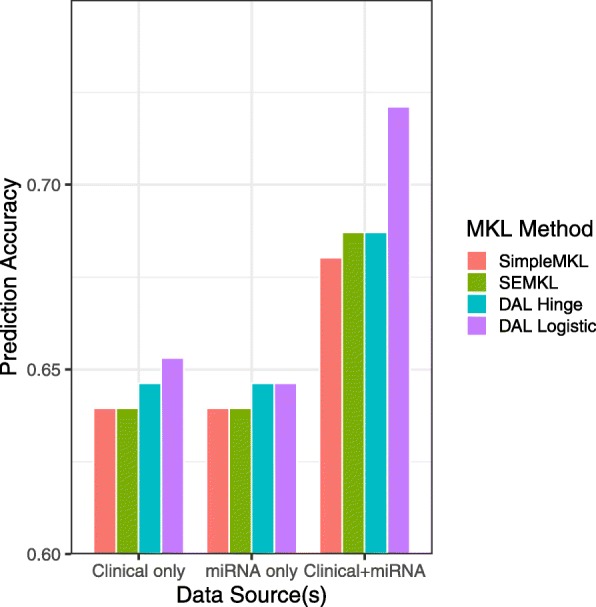
Prediction accuracy of MKL implementations using clinical and miRNA data individually and together in a single analysis using 198 patients to train each model and 85 patients to test the corresponding model. DAL Hinge and DAL Logistic refer to conducting DALMKL under different loss functions

There are 283 samples in this dataset. We used 70% (198) as the train samples and 30% (85) as a test set. For all kernel and variable prioritization, only the training set was used, and then the final classification accuracy of MKL was computed for the final MKL model. Candidates for the clinical kernels were constructed using kernels for stage and age, and the average of these two as a kernel. To avoid the curse of dimensionality, we include the 65 top-ranked genes, based on p-value from testing for differences in mean expression for patients who survived more than 3 years and those who did not. We used these 65 genes to conduct SVM with 10 fold cross-validation for many several radial kernels (*σ*=10^−10^,…,10^10^) to identify the range that leads to the highest predictive accuracy. Ultimately in our MKL analysis, we used a linear kernel, and 3 radial kernels with *σ*=10^−4^,10^−3^,10^−2^. Surprisingly, using miRNA data only has similar prediction accuracy as clinical information only, but using both data sources leads to a substantially higher accuracy than either of the individual data sources (Fig. 3).

### Hoadley data

Hoadley et al. (2018) conducted integrative molecular analyses for all tumors in TCGA [23]. We studied 15 cancer types which were selected because they had a total of greater than 300 tumors and more than 20 events. Survival was dichotomized by a cutoff that was selected such that the proportion of patients that survived was 0.4–0.6. Patients who were right-censored were not included in the analysis. Gene expression data was used to find relationships between gene sets and our binary survival outcome. In this analysis, we focused on 50 gene sets that are included in the hallmark gene sets introduced by Liberzon et al. (2015) [24], which represent specific well-defined biological states or processes. The cancer types and survival cutoff are provided in Additional file 1: Table S1 and S2.

To identify gene sets that may aid in classification, SVM is employed with cross-validation to identify which kernel shape and hyperparameter is most suitable. Gene sets were not considered if the training accuracy was less than the no information rate (*NIR*). This occurred when SVM classified all patients into one class, typically the largest class. The remaining gene sets were introduced to MKL using their shape and hyperparameter that leads to the highest accuracy in SVM. Details for how many gene sets were included for MKL are in Additional file 1: Table S3. Gene sets that have significant importance can be areas for future study.

**Fig. 4:**
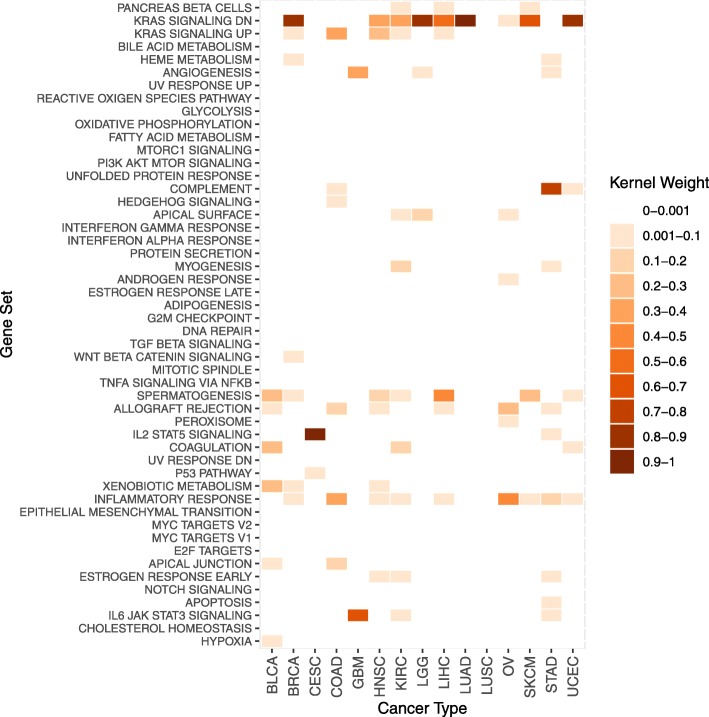
Heatmap of gene set importance for each of the 15 cancer types considered. (DALMKL Logistic)

In Fig. 4 and Additional file 1: Figure S3, we see that kernel weights are similar in SEMKL, and DALMKL (both hinge and logistic loss), while SimpleMKL is quite different and is often times less sparse than other methods. Additional file 1: Table S3 displays the prediction accuracy for each method. DALMKL tends to be the most accurate. There are cases, such as ovarian cancer (OV), where SimpleMKL allocates weight more evenly across the gene sets and can achieve a significant increase in accuracy. On the other hand, when all methods only consider a small number of gene sets SimpleMKL performs the worst.

The pan-cancer pathway analysis revealed multiple gene sets that carry important prognostic values. Interestingly, many pathways such as KRAS signaling, inflammatory response and spermatogenesis had non-zero kernel-based importance scores across many cancer types. We hope the finding will spur additional research into the role of these pathways in cancer development and prognosis especially spermatogenesis, which is less studied compared with other pathways in cancer.

## Conclusion

Integrating heterogeneous data sources into a single analysis allows patients to obtain more accurate prognoses or diagnoses. MKL can construct non-linear classification without any parametric assumptions for a single or multiple data types. Additionally, MKL may not suffer from overfitting because the final decision rule is based on a weighted average of SVM models. Kernel weights from MKL can have an appealing interpretation and help identify data sources that are most important in the classifier.

There are several considerations to be made regarding which MKL algorithm to use. If a small number of kernels are used, then each of the four methods seems to have similar performance. However, if a large number of kernels are used then DALMKL should be used. Regardless of the number of kernels, SimpleMKL and SEMKL have similar run times, however, DALMKL tends to run significantly faster. There are currently no recommendations for selection of cost parameter is SimpleMKL or SEMKL, while DALMKL provides recommendations and a formula to estimate a comparable cost for wrapper methods. DALMKL should be used first to get a range of cost values for SimpleMKL or SEMKL. A drawback of DALMKL is that the parameters for the optimization problem are more complicated and therefore not easy to interpret, while only a little bit of knowledge about SVM is needed to understand the parameters in SEMKL and SimpleMKL.

The real data analyses presented in this paper are biased, i.e. the censoring mechanism was completely ignored. Also, the selection of survival threshold was picked to provide an approximately even split of the binary outcomes potentially losing biological meaning. Extensions to MKL can be made to account for an imbalance of samples between the two groups, by modifying the objective function such that there is a different cost associated with misclassification for both classes. MKL presents opportunities to answer statistical questions. For instance, by considering different loss functions MKL can be extended to regression and survival analysis settings. The problem of missing data has been addressed for MKL [25] but there is still a lot of room for improvement. Utilizing kernel alignment is an additional step in kernel prioritization than can greatly increase the performance of MKL algorithms [21].

## Additional file

Additional file 1 This file contains a brief summary of SVM and MKL, 3 tables, and 1 figure that better summarize the experiment with Hoadley data. (PDF 376 kb)

## Data Availability

**Project name:** RMKL **Project home page:** https://CRAN.R-project.org/package=RMKL **Operating system:** Platform independent **Programming language:** R **Other requirements:** **License:** GPL-3

## Abbreviations

DALMKL: Dual augmented lagrangian mkl
MKL: Multiple kernel learning
SEMKL: Simple and efficient MKL
SimpleMKL: Simple MKL
SVM: Support vector machine
TCGA: The cancer genome atlas
