# Supplementary material for: Multiple-kernel learning for genomic data mining and prediction
Source: BMC Bioinformatics. 2019 Aug 15;20:426. doi: 10.1186/s12859-019-2992-1 (PMC6694479; doi:10.1186/s12859-019-2992-1)
Supplement: Supplementary file 1 — This file contains a brief summary of SVM and MKL, 3 tables, and 1 figure that better summarize the experiment with Hoadley data. (PDF 376 kb) [file 12859_2019_2992_MOESM1_ESM.pdf]

1 Supplemental Material

1.1 Support Vector Machine

We will be considering samples (x_i, y_i), where $y = \pm 1$ and the vector of covariates $x \in \mathcal{X}$. The goal of SVM is to find the hyperplane, $\{w : w \cdot x + b\}$, that correctly separates the data into two classes and has the largest possible distance between the two groups, which is referred to as margin. The SVM classification rule is defined to be $y(x) = w \cdot x + b$. The SVM problem can be expressed as the following convex optimization problem:

$$\begin{aligned} &\text{minimize } ||w||^2 \\ &\text{subject to } y_i(w \cdot x_i + b) \geq 1, \; i = 1, \ldots, n \end{aligned} \tag{1}$$

Note that $y_i(w \cdot x_i + b)$ is greater than 1 if y_i and $w \cdot x_i + b$ have the same sign, i.e. the sample is correctly classified. This problem is known as the hard margin formulation and is only feasible when two groups can be perfectly separated by a linear function [1].

It is rare that data are perfectly linearly separable. We can relax (1) so that samples are allowed to be misclassified, by incorporating a penalty for samples that misclassified. The following optimization convex problem is referred to as the soft margin problem:

$$\begin{aligned} &\text{minimize } \frac{1}{2}||w||^2 + C\sum_{i=1}^{n}\xi_i \\ &\text{subject to } y_i(w \cdot x_i + b) \geq 1 - \xi_i \; i = 1, \ldots, n \\ &\qquad \xi_i \geq 0, \end{aligned} \tag{2}$$

where ξ_i are slack variables. Commonly we use $\xi_i = \max(0, y_i(w \cdot x_i + b))$, which is known as the hinge loss function. The parameter C controls the penalty of misclassification, and a value for C is typically found via cross-validation. Larger values of C can lead to a smaller margin to minimize the misclassifications, while smaller values of C may produce a larger margin that can lead to more misclassifications (illustrated in Supplemental Figure 1). Problem (2) is typically not solved directly, but rather by solving the Lagrangian dual [1].

The Lagrangian is the sum of the original objective function and a term that involves the constraints and multiplier. The Lagrangian of (2) is given below:

$$\begin{aligned} \mathcal{L}(w, \xi, \alpha, b) = &\frac{1}{2}||w|| + C\sum_{i=1}^{n}\xi_i \\ &+ \sum_{i=1}^{n}\alpha_i(1 - \xi_i - y_i(w \cdot x_i + b)) - \sum_{i=1}^{n}\mu_i\xi_i \end{aligned} \tag{3}$$

where $\alpha_i, \mu_i \geq 0$. The minimizers of $\mathcal{L}$ are found by setting the gradient of $\mathcal{L}$ equal to zero and

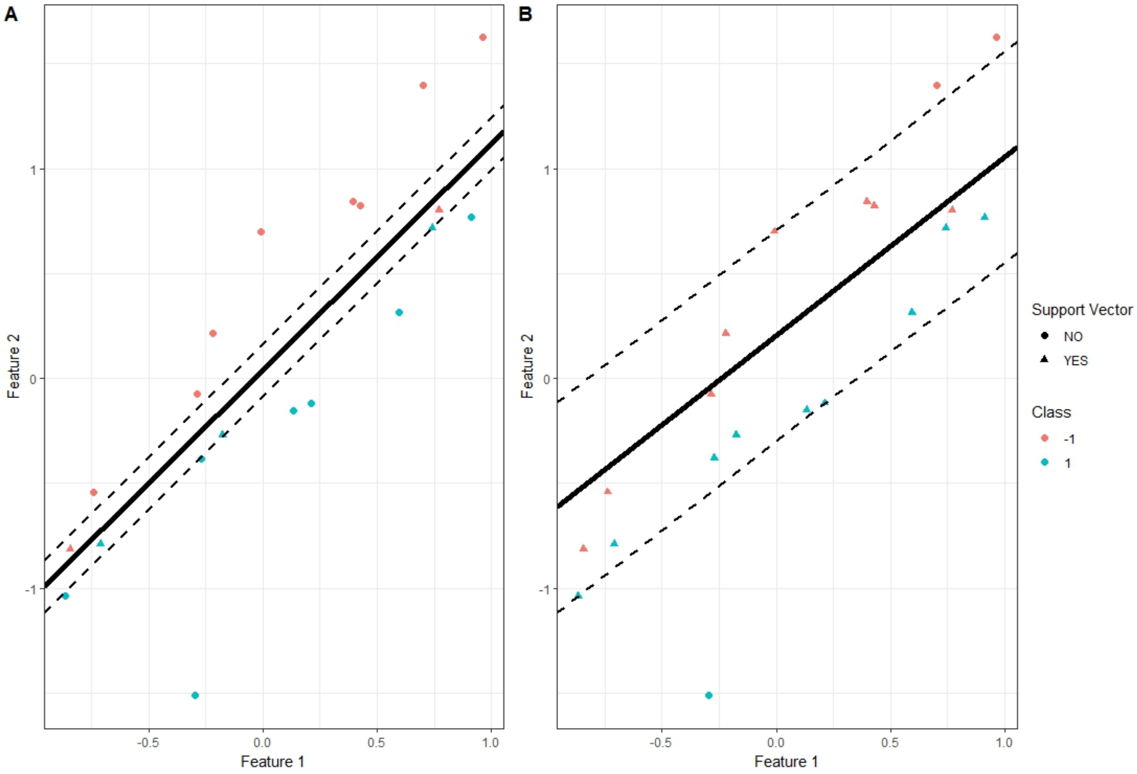

Supplemental Figure 1: Illustration of trade off between high and low values of C in (2).

solving the resulting system of equations:

$$\frac{\partial \mathcal{L}}{\partial w} = w - \sum_{i=1}^{n} \alpha_i y_i x_i \overset{set}{=} 0, \tag{4a}$$

$$\frac{\partial \mathcal{L}}{\partial b} = \sum_{i=1}^{n} \alpha_i y_i \overset{set}{=} 0, \text{ and} \tag{4b}$$

$$\frac{\partial \mathcal{L}}{\partial \xi_i} = C - \alpha_i - \mu_i \overset{set}{=} 0, \; i = 1, \ldots, n, . \tag{4c}$$

Equations (4b) and (4c) provide two new constraints, namely $\sum_{i=1}^{n} \alpha_i y_i = 0$ and $\alpha_i \leq C - \mu_i$, and provides a representation for the optimal hyperplane $w = \sum_{i=1}^{n} \alpha_i y_i x_i$. Plugging the solutions of (4)

into the Lagrangian dual of (3) yields:

$$\begin{aligned} \text{maximize } & \sum_{i=1}^{n} \alpha_i \\ & - \frac{1}{2} \sum_{i=1}^{n} \sum_{i'=1}^{n} (\alpha_i y_i)(x_i \cdot x_{i'})(\alpha_{i'} y_{i'}) \\ \text{subject to } & \sum_{i=1}^{n} \alpha_i y_i = 0 \\ & 0 \leq \alpha_i \leq C,\ i = 1, \ldots, n, \end{aligned} \tag{5}$$

where $(x_i \cdot x_{i'})$ denotes the dot product between x_i and $x_{i'}$. This problem is a quadratic programming problem that can solved with many solvers and can be solved much more efficiently than (2). The Karush-Kuhn-Tucker (KKT) conditions are necessary conditions for solving a non-linear programming problem and for SVM these conditions are (4a)-(4c), and:

$$y_i(w \cdot x_i + b) \geq 1 - \xi_i \tag{6a}$$

$$\xi_i \geq 0 \tag{6b}$$

$$\alpha_i[(w \cdot x_i + b) - (1 - \xi_i)] = 0 \ \forall \ i \tag{6c}$$

$$\mu_i \epsilon_i = 0 \ \forall \ i, \tag{6d}$$

which lead to the following conditions for the support vectors,

$$\alpha_i = 0 \Rightarrow y_i(w \cdot x_i) \geq 1 \text{ and } \xi_i = 0, \tag{7a}$$

$$0 < \alpha_i < C \Rightarrow y_i(w \cdot x_i) = 1 \text{ and } \xi_i = 0, \tag{7b}$$

$$\alpha_i = C \Rightarrow y_i(w \cdot x_i) \leq 1 \text{ and } \xi_i > 0. \tag{7c}$$

The resulting classification function produced by SVM algorithms is computed with samples satisfying $0 < \alpha_i < C$, which correspond to points that are on the margin. These points are called support vectors and the number of support vectors is typically much smaller than the number of samples which helps make SVM algorithms faster [2].

Kernels can be employed to map data into a higher dimensional feature space where the data are linearly separated. A kernel function $K : \mathcal{X} \times \mathcal{X} \rightarrow \mathbb{R}$ that for all $x_i, x_{i'}$ that satisfies $K(x_i, x_{i'}) := (\phi(x_i) \cdot \phi(x_{i'}))$ where $\phi : \mathcal{X} \rightarrow \mathcal{H}$, and $\mathcal{H}$ is a Hilbert space. Kernel functions are different similarity measures between samples and K is a symmetric positive definite matrix. The above derivation can be extended to non-linear classification by simply replacing $w \cdot x$ and $x \cdot x'$, in (2) and (3), $f(x) = K(w, x)$ and $K(x, x')$,

$$\begin{aligned} \text{minimize } & \frac{1}{2}||f||^2 + C \sum_{i=1}^{n} \xi_i \\ \text{subject to } & y_i(f(x_i) + b) \geq 1 - \xi_i,\ i = 1, \ldots, n, \end{aligned} \tag{8}$$

The Lagrangian dual can be constructed in a similar fashion as above, of (8) which yields:

$$
\begin{aligned}
\text{maximize } & \sum_{i=1}^{n} \alpha_i \\
& - \frac{1}{2} \sum_{i=1}^{n} \sum_{i'=1}^{n} (\alpha_i y_i) K(x_i, x_{i'}) (\alpha_{i'} y_{i'}) \\
\text{subject to } & \sum_{i=1}^{n} \alpha_i y_i = 0 \\
& 0 \leq \alpha_i \leq C \text{ for all } i = 1, \ldots, n.
\end{aligned} \tag{9}
$$

Since K is a symmetric positive definite matrix, (9) is a quadratic programming problem. Unlike SVM, there is typically not a closed form expression for $f(x)$, thus it is difficult to interpret the results [1],[2].

1.2 Multiple Kernel Learning

It has been shown that the convex combinations of kernel functions is a kernel function. An avenue for improvement is to utilize several different representations of the data and allow an algorithm to use a weighted average of these representations of the data. This can help automate kernel prioritization by using a combination of kernel functions for a set of candidate kernels, this is the main idea of multiple kernel learning (MKL). Combining kernels is possible by decomposing the input space into blocks as follows $\mathcal{X} = \mathcal{X}_1 \times \cdots \times \mathcal{X}_m$ where each sample can be expressed as $\phi(x_i) = (\phi_i(x_{i1}), \ldots, \phi_i(x_{im}))$. MKL can be formulated as the following optimization problem:

$$
\begin{aligned}
\text{minimize } & \frac{1}{2} \left(\sum_{j=1}^{m} \gamma_j^{-1} ||f_j||_{\mathcal{H}_j} \right)^2 + C \sum_{i=1}^{n} \xi_i \\
\text{subject to } & y_i \left(\sum_{j=1}^{m} f_j(x_i) + b \right) \geq 1 - \xi_i, \; i = 1, \ldots, n \\
& \sum_{j=1}^{n} \gamma_j = 1, \; \gamma_j \geq 0.
\end{aligned} \tag{10}
$$

This problem remains convex, however it is not smooth which leads to computational issues. Using the same procedure as before, the Lagrangian dual given below:

$$\begin{aligned} \underset{\gamma}{\text{minimize}}\ \underset{\alpha}{\text{maximize}}\ & \frac{1}{2}\sum_{j=1}^{m}\gamma_j(\alpha y)^T K_j(\alpha y) \\ & -\sum_{i=1}^{n}\alpha_i \\ \text{subject to}\ & \sum_{i=1}^{n}\alpha_i y_i = 0 \\ & 0 \le \alpha_i \le C,\ i = 1,\dots,n \\ & \sum_{j=1}^{m}\gamma_j = 1 \\ & \gamma_j \ge 0, j = 1,\dots,m, \end{aligned} \tag{11}$$

where γ_i is the weight of the ith kernel. MKL allows the flexibility to assign kernels on an individual variables basis, or as a data integration tool by assigning the different kernels to multiple data sources [3].

There have been many algorithms proposed to conduct MKL. One class of MKL algorithms are wrapper methods which iteratively solve a single kernel learning problem for a given combination of kernel weights. Wrapper methods iteratively optimize $f,\ b,\ \alpha$ with the kernel weights fixed, sometimes referred to as the fixed weights problem, and then optimize the kernel weights with $f,\ b,\ \alpha$ fixed. A characteristic of wrapper methods is that they reformulate either the dual or primal of the MKL problem to use off-the-shelf efficient solvers. A shortcoming of wrapper methods is the optimization of $f,\ b,\ \alpha$ is inefficient, and maybe unnecessary if the kernel weights are not optimal [4].

SimpleMKL uses gradient descent to solve for the direction that has the most improvement. Then uses lines search to find the optimal kernel weights, with fixed f_j, b, and ξ. For each candidate γ, SimpleMKL must solve an SVM problem [3]. Though wrapper methods are easy to implement, they may have poor convergence or produce solutions that are far from the global optimum. SimpleMKL updates γ using gradient descent, SEMKL directly computes γ by

$$\gamma_i = \frac{||f_j||_{]\mathcal{H}_j}}{\sum_k ||f_k||} = \frac{\alpha K_j \alpha}{\sum_k \alpha K_k \alpha}. \tag{12}$$

Smaller γ_j corresponds to smooth f_j, and larger γ_j correspond to more noisy f_j [5].

DALMKL optimizes the dual augmented Lagrangian of a proximal formulation of the MKL problem. This formulation presents a unique set of problems such as the conjugate of a loss function must have no non-differentiable points in the interior of its domain and cannot have a finite gradient at the boundary of its domain. Additional primal variables are added so that $\phi_\gamma(\cdot, \alpha, b)$ becomes differentiable. The inner function (14) is differentiable and the gradient and Hessian only depend on the active kernels making gradient descent efficient. Though DALMKL and wrapper function attempt to construct a kernel using a convex combination of kernels, they parameterizations are quite different. Suzuki points this out for the C parameter in particular stating the following

relationship

$$\tilde{C} = C\left(\sum_{j=1}^{m} ||f_j^*||_{\mathcal{H}_j}\right) \tag{13}$$

Suzuki recommends a range of $\tilde{C}$ =0.5, 0.05, and 0.005, while there is no clear recommendations C for wrapper methods. Below is a more formal derivation of DALMKL[6].

DALMKL was formulated to utilize the block 1-norm proposed by Bach [7]. The block 1-norm problem is solved by optimizing the dual augmented-Lagrangian. Consider the following general formulation for MKL notation:

$$\min\left\{L\left(\bar{K}_j\alpha_j + b\mathbf{1}\right) + \phi_C(\alpha)\right\}, \tag{14}$$

where $\bar{K}\alpha = \sum_{j=1}^{m} K_j\alpha_j$, and $\phi_C(\alpha) = C\sum_{j=1}^{m} ||\alpha_j||_{K_j}$. A non-decreasing sequence $\Omega = \{\omega^{(1)}, \omega^{(2)}, \dots\}$ are introduced to allow to (15) as follows:

$$\begin{aligned}\min\Big\{&L\left(\bar{K}_j\alpha_j + b\mathbf{1}\right)\\ &+ \phi_C(\alpha) + \frac{||\alpha - \alpha^{(t)}||^2}{2\omega^{(t)}} + \frac{(b - b^{(t)})^2}{2\omega^{(t)}}\Big\},\end{aligned} \tag{15}$$

where $\alpha^{(t)}$ and $b^{(t)}$ are the tth updated values of α and b. This problem is referred to as the proximal MKL problem. The constraint $z = \bar{K}\alpha + b\mathbf{1}$ is added (14) and the resulting Lagrangian dual is

$$\begin{aligned}L(z) + \phi_C(\alpha) &+ \frac{||\alpha - \alpha^{(t)}||^2}{2\omega^{(t)}}\\ &+ \frac{(b - b^{(t)})^2}{2\omega^{(t)}} + \rho^T(z - \bar{K}\alpha - b\mathbf{1}).\end{aligned} \tag{16}$$

Minimizing (16) with respect to (z, α, b) results in

$$\begin{aligned}&\min_z -L^*(-\rho)\\ &+ \frac{1}{2\omega^{(t)}}\sum_{j=1}^{m}\left|\left|\text{prox}(\alpha_j^{(t)} + \omega^{(t)}\rho|\phi_{\omega^{(t)}C}(\alpha))\right|\right|_{K_j}^2\\ &- \frac{1}{2\omega^{(t)}}\left(b^{(t)} + \omega^{(t)}\sum_{i=1}^{n}\rho_i\right)^2,\end{aligned} \tag{17}$$

where L^* is the convex conjugate of L, and

$$\text{prox}(v_j|\phi_C^{(m)}) = \begin{cases}0 & \text{if } ||v_m||_{K_m} \leq C\\ \frac{||v_m||_{K_m} - C}{||v_m||_{K_m}}v_m & \text{otherwise}\end{cases}. \tag{18}$$

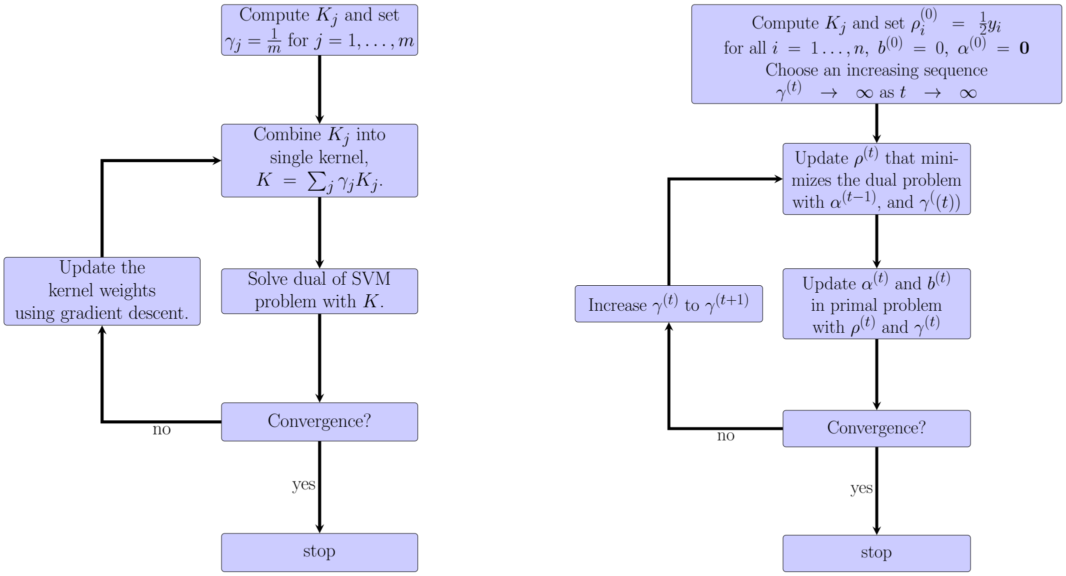

Supplemental Figure 2: Workflow for general wrapper (left) and DALMKL (right) implementations of MKL

We optimize (16) in two cycles. In the inner cycle we optimize the dual vector ρ by

$$\begin{aligned} \rho^{(t)} &= \operatorname{argmin}_\rho \left\{ L^* \left(-\rho \right) \right\} \\ &+ \frac{1}{2\gamma^{(t)}} \sum_{j=1}^{m} \left\| \operatorname{prox} \left(\alpha_j^{(t-1)} + \gamma^{(t)} \rho | \phi^{(i)}_{\gamma^{(t)} C} \right) \right\|_{K_j}^2 \\ &+ \left(b^{(t-1)} + \gamma^{(t)} \sum_{j=1}^{n} \rho_j \right) \end{aligned} \quad (19)$$

and in the outer cycle we update $\alpha^{(t)}$ and $b^{(t)}$ with $\alpha^{(t)} = \operatorname{prox} \left(\alpha_j^{(t-1)} + \gamma^{(t)} \rho^{(t)} | \phi^{(i)}_{\gamma^{(t)} C} \right)$ and $b^{(t)} = b^{(t-1)} + \gamma^{(t)} \sum_{i=1}^{n} \rho_i^{(t)}$. The paper provides a detailed overview of the derivation and outlines many scenarios that use elastic net and block $q-$norm, for $q \geq 1$, regularizations, as well as, logistic, squared, hinge, and $\epsilon-$ loss functions. These scenarios make DALMKL a promising method for extension to other employed in other arenas such as causal inference, or survival analysis.

1.3 Summary of Hoadley Data

Cancer types listed in Supplemental Table 1 were selected because there were more than 300 samples before removal of censored patients. Prostate Adenocarcinoma (PRAD) and Thyroid Carcinoma (THCA) were not considered because they had 10 and 16 deaths before the end of followup. Survival outcome was dichotomized by a threshold that the survival rate was between $[0.4, 0.6]$. Supplemental Table 2 provides a summary of data for each cancer type.

Full Name	TCGA Abbreviation
Bladder Urothelial Carcinoma	BLCA
Breast invasive carcinoma	BRCA
Cervical Squamous Cell Carcinoma and Endocervical Adenocarcinoma	CESC
Colon Adenocarcinoma	COAD
Glioblastoma multiforme	GBM
Head and Neck squamous cell carcinoma	HNSC
Kidney renal clear cell carcinoma	KIRC
Brain Lower Grade Glioma	LGG
Liver Hepatocellular Carcinoma	LIHC
Lung Adenocarcinoma	LUAD
Lung Squamous Cell Carcinoma	LUSC
Ovarian serous cystadenocarcinoma	OV
Skin Cutaneous Melanoma	SKCM
Stomach Adenocarcinoma	STAD
Uterine Corpus Endometrial Carcinoma	UCEC

Supplemental Table 1: Cancer types and their TCGA abbreviations.

Cancer Type	Survival Threshold	Survived Longer than Threshold	Died Prior to Threshold	Total	$\hat{p}_1$	n_{train}	n_{test}
BLCA	2 years	139	142	281	0.489	196	85
BRCA	7 years	141	120	261	0.540	182	79
CESC	4 years	61	62	123	0.495	86	37
COAD	3 years	110	77	187	0.588	130	57
GBM	1 year	73	58	131	0.595	91	40
HNSC	3 year	144	148	292	0.493	204	88
KIRC	4.5 years	175	145	320	0.547	224	96
LGG	5 years	90	90	180	0.5	126	54
LIHC	2.5 years	108	101	209	0.517	146	63
LUAD	3 years	133	137	270	0.492	189	81
LUSC	2.5 years	190	138	328	0.579	229	99
OV	3 years	128	96	224	0.571	158	66
SKCM	5 years	152	144	296	0.513	207	89
STAD	2 years	108	134	242	0.440	169	73
UCEC	5 years	105	79	184	0.570	128	56

Supplemental Table 2: Summary of patients with each cancer type. Here $\hat{p}_1$ is the proportion of patients that survived longer than the threshold. $NIR = \max(p_{1,test}, p_{-1,test})$, where $p_{i,test}$ is the proportion of patients with outcome i in the test set.

1.4 Results

Cancer Type	Number of Gene Sets	SimpleMKL	SEMKL	DAL Hinge	DAL Logistic	NIR
BLCA	27	0.635	0.635	0.659	0.635	0.565
BRCA	50	0.551	0.573	0.570	0.557	0.506
CESC	48	0.676	0.757	0.757	0.811	0.676
COAD	40	0.632	0.632	0.596	0.632	0.596
GBM	48	0.675	0.725	0.725	0.725	0.600
HNSC	50	0.691	0.680	0.619	0.670	0.536
KIRC	50	0.729	0.698	0.677	0.698	0.573
LGG	50	0.818	0.782	0.800	0.800	0.527
LIHC	50	0.667	0.651	0.714	0.683	0.524
LUAD	2	0.593	0.630	0.605	0.605	0.543
LUSC	27	0.535	0.535	0.576	0.545	0.535
OV	9	0.667	0.621	0.636	0.636	0.500
SKCM	50	0.573	0.607	0.562	0.618	0.517
STAD	4	0.616	0.644	0.589	0.630	0.534
UCEC	8	0.719	0.684	0.772	0.702	0.667

Supplemental Table 3: Prediction accuracy for each of the four methods and each cancer type, and the number of genes sets (kernels) considered for MKL. Values in red correspond to the most accurate method and values blue correspond to least accurate method.

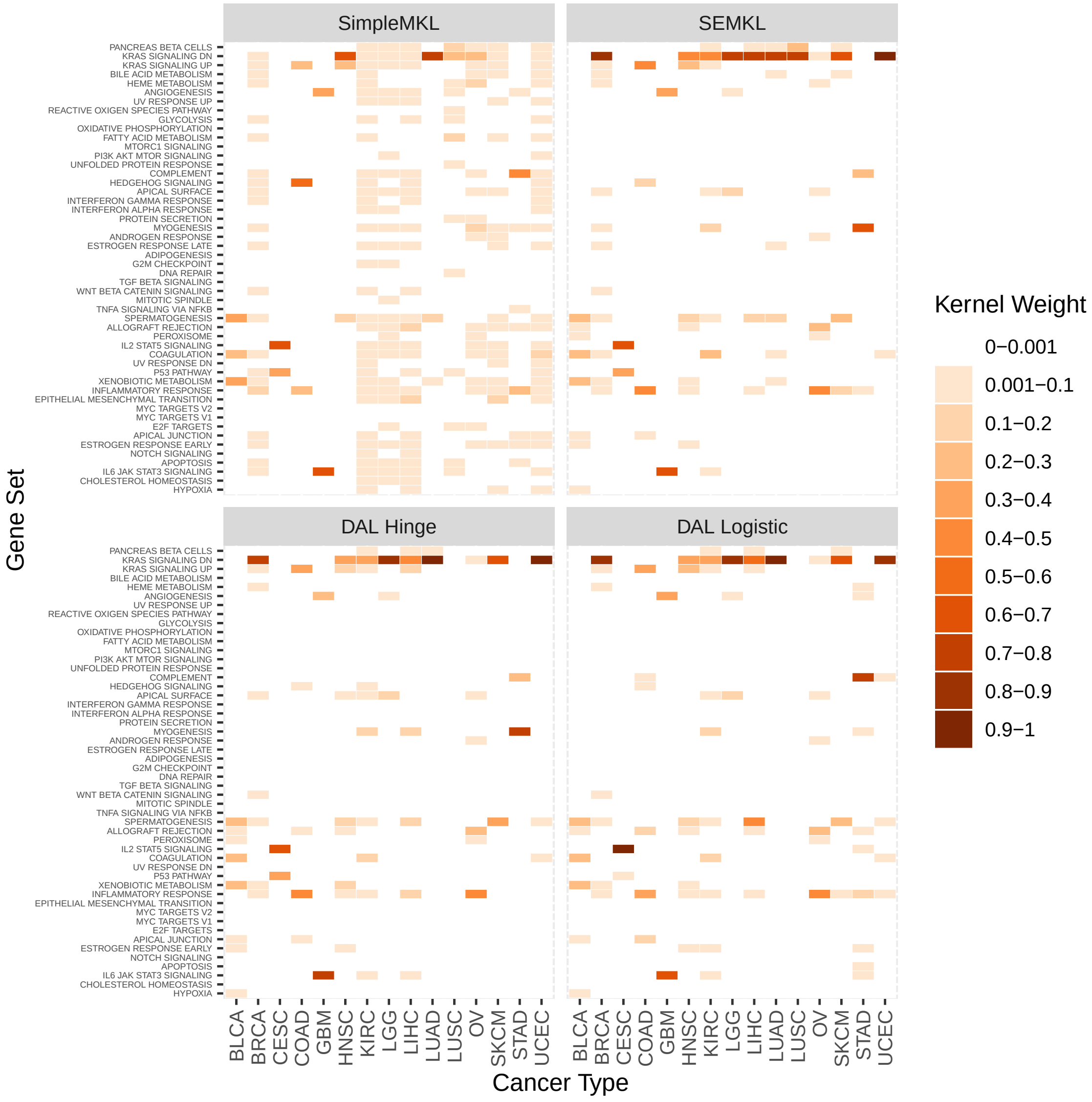

Supplemental Figure 3: Heatmap of the kernel importance for each method and cancer type.

References

[1] Cortes C, Vapnik V. Support-Vector Networks. Machine Learning. 1995;20(3):273–297.

[2] Hastie T, Tibshirani R, Friedman J. The Elements of Statistical Learning. Springer Series in Statistics. New York, NY, USA: Springer New York Inc.; 2001.

[3] Rakotomamonjy A, Bach F, Canu S, Grandvalet Y. SimpleMKL. Journal of Machine Learning Research. 2008 11;9:2491–2521.

[4] Sonnenburg S, Rätsch G, Schäfer C, Schölkopf B. Large Scale Multiple Kernel Learning. J Mach Learn Res. 2006 Dec;7:1531–1565. Available from: http://dl.acm.org/citation.cfm?id=1248547.1248604.

[5] Xu Z, Jin R, Yang H, King I, Lyu MR. Simple and Efficient Multiple Kernel Learning by Group Lasso. In: Proceedings of the 27th International Conference on International Conference on Machine Learning. ICML'10. USA: Omnipress; 2010. p. 1175–1182. Available from: http://dl.acm.org/citation.cfm?id=3104322.3104471.

[6] Suzuki T, Tomioka R. SpicyMKL: a fast algorithm for Multiple Kernel Learning with thousands of kernels. Machine Learning. 2011;85(1):77–108.

[7] Bach FR. Consistency of the Group Lasso and Multiple Kernel Learning. J Mach Learn Res. 2008 Jun;9:1179–1225. Available from: http://dl.acm.org/citation.cfm?id=1390681.1390721.
